# 

**Published:** 1871-12

**Authors:** 


					﻿VOL. XII. NOV. AND DEC., 1871. NOS. 11 & 12.
CHICAGO
MEDICAL EXAMINER
N. S. DAVIS, M. D., Editor.
AND
F. H DAVIS, M. D., Assistant Editor.
FERMS' $3-00 rblR	IJftP MüV.'LYCE
ANN ARBOR:
COURIER STEAM PRINTING HOUSE.
187 1.
				

